# Accelerated epigenetic age, inflammation, and gene expression in lung: comparisons of smokers and vapers with non-smokers

**DOI:** 10.1186/s13148-023-01577-8

**Published:** 2023-10-11

**Authors:** Min-Ae Song, Kellie M. Mori, Joseph P. McElroy, Jo L. Freudenheim, Daniel Y. Weng, Sarah A. Reisinger, Theodore M. Brasky, Mark D. Wewers, Peter G. Shields

**Affiliations:** 1 Division of Environmental Health Sciences, College of Public Health, The Ohio State University, 404 Cunz Hall, 1841 Neil Ave., Columbus, OH 43210 USA; 2 Department of Biomedical Informatics, College of Medicine, The Ohio State University, Columbus, OH USA; 3 Comprehensive Cancer Center, The Ohio State University and James Cancer Hospital, Columbus, OH USA; 4 Department of Epidemiology and Environmental Health, School of Public Health and Health Professions, University at Buffalo, Buffalo, NY USA

**Keywords:** Epigenetic aging, Biological aging, Vaping, Smoking, Gene expression, Inflammation

## Abstract

**Background:**

Cigarette smoking and aging are the main risk factors for pulmonary diseases, including cancer. Epigenetic aging may explain the relationship between smoking, electronic cigarette vaping, and pulmonary health. No study has examined smoking and vaping-related epigenetic aging in relation to lung biomarkers.

**Methods:**

Lung epigenetic aging measured by DNA methylation (mAge) and its acceleration (mAA) was assessed in young (age 21–30) electronic cigarette vapers (EC, *n* = 14, including 3 never-smoking EC), smokers (SM, *n* = 16), and non-EC/non-SM (NS, *n* = 39). We investigated relationships of mAge estimates with chronological age (Horvath-mAge), lifespan/mortality (Grim-mAge), telomere length (TL-mAge), smoking/EC history, urinary biomarkers, lung cytokines, and transcriptome.

**Results:**

Compared to NS, EC and SM had significantly older Grim-mAge, shorter TL-mAge, significantly accelerated Grim-mAge and decelerated TL-mAge. Among SM, Grim-mAA was associated with nicotine intake and 4-(methylnitrosamino)-1-(3-pyridyl)-1-butanol (NNAL). For EC, Horvath-mAA was significantly correlated with puffs per day. Overall, cytokines (IL-1β, IL-6, and IL-8) and 759 transcripts (651 unique genes) were significantly associated with Grim-mAA. Grim-mAA-associated genes were highly enriched in immune-related pathways and genes that play a role in the morphology and structures of cells/tissues.

**Conclusions:**

Faster lung mAge for SM is consistent with prior studies of blood. Faster lung mAge for EC compared to NS indicates possible adverse pulmonary effects of EC on biological aging. Our findings support further research, particularly on epigenetic markers, on effects of smoking and vaping on pulmonary health. Given that most EC are former smokers, further study is needed to understand unique effects of electronic cigarettes on biological aging.

**Supplementary Information:**

The online version contains supplementary material available at 10.1186/s13148-023-01577-8.

## Background

While tobacco consumption has been falling in recent decades, cigarette smoking is still by far the leading preventable risk factor for lung cancer [1]. Of emerging tobacco products, electronic cigarettes have been marketed as a safe alternative to combustible cigarette smoking and may foster smoking cessation [2]. The major constituents of electronic cigarette liquids, including propylene glycol (PG) and vegetable glycerine (VG) as solvent carriers, are considered safe by the Food and Drug Administration (FDA) when used in foods and cosmetics. However, the long-term effects on the lung when these constituents are heated and inhaled during vaping are largely unknown.

Smoking-related disease pathogenesis involves multiple processes, including accelerating the process of organ aging and declining lung function [3–5]. Smoking is associated with biological aging indicated by telomere length [6], and lung aging is a critical risk factor for lung diseases and cancer, causing structural and physiological changes.

DNA methylation is an epigenetic marker that reflects recent and longer-term tobacco smoke exposure and is reversible after smoking cessation [7–11]. Studies, including ours [12], show smoking-related methylation enrichment in genes related to lung function, lung diseases, and cancer, including xenobiotic pathways, oxidative stress, and inflammation [7, 13–15]. While altered methylation has been well-studied for cigarette smoking [16–19], there is relatively little understanding of its association with electronic cigarettes in humans [12, 20–23].

DNA methylation age (mAge), known as “epigenetic aging,” has drawn significant attention as a tool for understanding age-related diseases [24–27]. Given that advancing age is the most important key risk factor for many cancers, including lung cancer [28], accelerated mAge reflecting faster biological aging in young individuals may indicate adverse health outcomes later in life. The discrepancy between mAge and chronological age is defined as mAge acceleration (mAA). mAA has been reported in comparisons of smokers to never-smokers in blood samples [24, 29–33]. Blood epigenetic aging (i.e., Grim-mAge) is suggested to be one of the biological mechanisms linking lifetime exposure to smoking and death in later life [34]. However, effects are unknown of electronic cigarette vaping on the lung, as the target organ, on epigenetic aging.

Given that mAge may be an important pathway to explain the association between smoking, electronic cigarette vaping, and target organ pathological effects, we investigated epigenetic aging in the lungs of smokers (SM) and electronic cigarette users (EC) compared to never-smokers (NS). We utilized three well-studied mAge estimates, Horvath-mAge for chronological aging [35], Grim-mAge for lifespan and mortality [36], and TL-mAge for telomere length [31]. Further, we examined the relations of mAge acceleration in lung with other biomarkers, including inflammation and gene expression and explored their potential implications in age-related pulmonary diseases, including lung cancer and chronic obstructive pulmonary disease (COPD).

## Results

### Assessment of DNA methylation age estimates and their accelerations in relation to chronological age in lungs

**Fig. 1:**
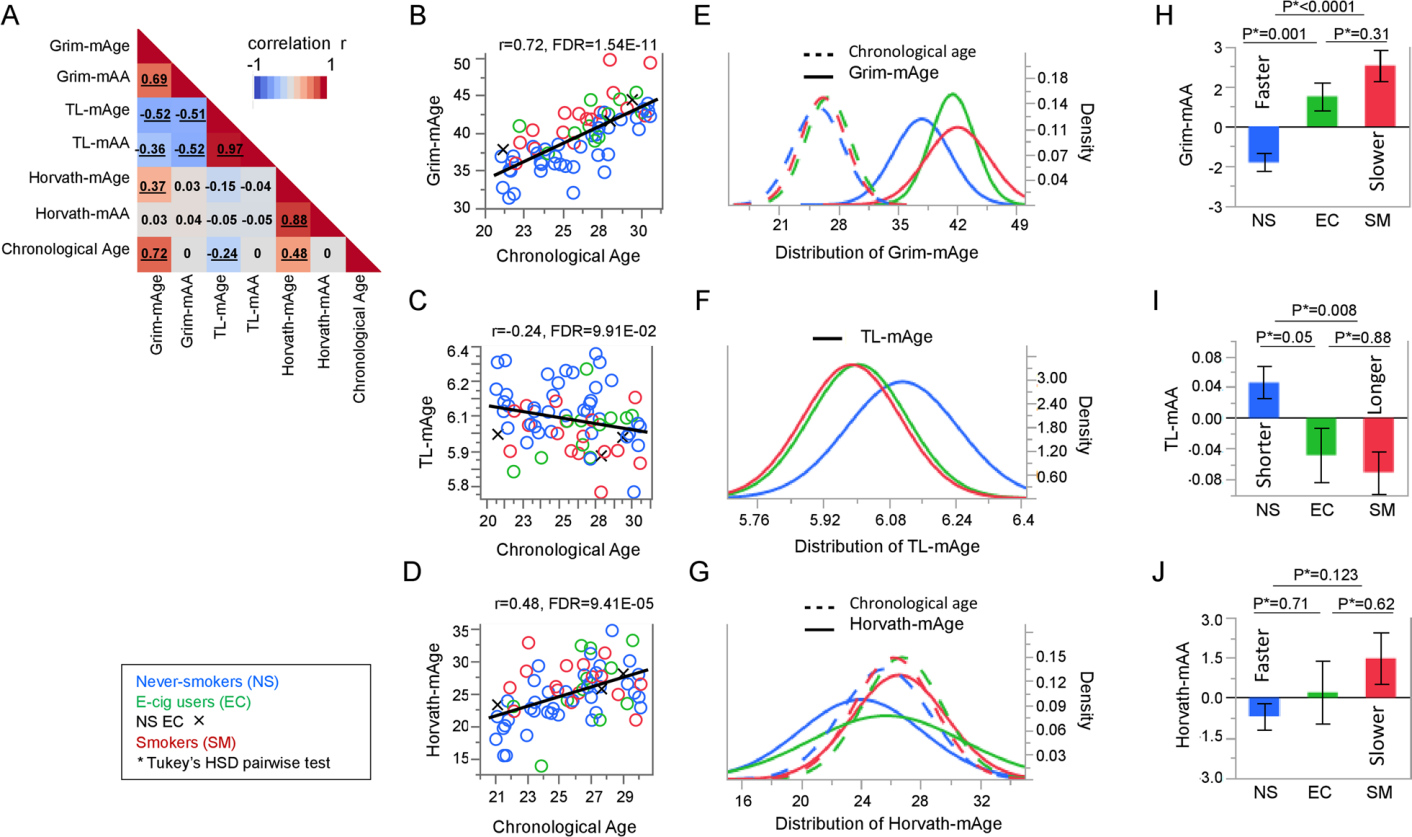
Correlations of between chronological age, methylation age (mAge), and accelerated mAge (mAA) estimates and associations with smoking and vaping status. **A** Pearson correlation plots displaying the associations between chronological age, mAge, and mAA measures. The scale bar displays the correlation coefficients (r) ranging from − 1 (blue) to 1 (red). **B–D** Correlation plots of associations between chronological age (x-axis) and mAge estimates (y-axis) for **B** Grim-mAge, **C** TL-mAge, and **D** Horvath-mAge. Each open dot represents individual never-smokers (NS, blue), electronic cigarette vapers (EC, green), and cigarette smokers (SM, red). Black X’s represent never-smoking EC (NS EC). **B**–**D** The line displayed reflects the linear regression line. **E–G** Plots with fitted normal curves displaying the age range (x-axis) and density of each given age value (y-axis). Each solid line represents the **E** Grim-mAge, **F** TL-mAge or **G** Horvath-mAge, for NS (blue), EC (green), or SM (red), while each dotted line represents chronological age for NS (blue), EC (green) or SM (red). Histograms of raw values (non-fitted) in Additional file 1: Fig. 1. **H–J** Bar charts comparing mean mAA estimates (y-axis) between NS (blue), EC (green), and SM (red) (x-axis) for **H** Grim-mAA, **I** TL-mAA, and **J** Horvath-mAA. Error bars display the standard error. P-values by Tukey’s HSD pairwise test are provided

To investigate mAge in lungs of healthy EC, SM, and NS, we calculated Horvath-mAge, Grim-mAge, and TL-mAge, and their acceleration (faster or slower mAge, -mAA), including Horvath-mAA, Grim-mAA, and TL-mAA. Chronological age was significantly positively correlated with Grim-mAge (r = 0.72, FDR = 1.54E−11) and Horvath-mAge (r = 0.48, FDR = 9.41E−05), while significantly negatively correlated with TL-mAge (r = − 0.24, FDR = 9.91E−02) (Fig. 1A–D). None of the mAA estimates were significantly correlated with chronological age. Comprehensive correlations between mAge estimates, their accelerations, and chronological age for EC, SM, and NS are provided in Additional file 2: Table 1**.**

### Associations of lung DNA methylation age estimates and their accelerations between smokers, electronic cigarette users, and never-smokers

All three mAge estimates were significantly different in SM compared to NS (Table 1). Additionally, when comparing EC to NS and SM, EC had significantly older Grim-mAge (41.47 vs. 37.85, Tukey’s HSD test P = 0.002, Fig. 1E) and shorter TL-mAge (6.01 vs. 6.11, Tukey’s HSD test P = 0.02, Fig. 1F) compared to NS, but similar to SM (42.07, Tukey’s HSD test P = 0.88, for Grim-mAge and 5.99, Tukey’s HSD test P = 0.94 for TL-mAge) (Table 2). SM did not have significantly different Horvath-mAge compared to NS (Tukey’s HSD test P = 0.10) and EC (Tukey’s HSD test P = 0.84) (Table 1, Fig. 1G).

**Table 1:** Associations of mAge measures with smoking group

	Mean (SD)				Tukey HSD testing, P		
	All participants (*n* = 69)	NS (*n* = 39)	EC (*n* = 14)	SM (*n* = 16)	EC versus NS	SM versus NS	SM versus EC
Chronological age	26 (2.8)	25.6 (3.0)	26.8 (2.7)	26.2 (2.7)	0.40	0.77	0.85
*mAge*							
Grim-mAge	39.56 (3.82)	37.85 (3.36)	41.47 (2.61)	42.07 (3.73)	**0.002**	**0.0002**	0.88
TL-mAge	6.06 (0.14)	6.11 (0.14)	6.01 (0.12)	5.99 (0.12)	**0.02**	**0.006**	0.94
Horvath-mAge	24.95 (4.23)	24.01 (4.10)	25.72 (5.13)	26.59 (3.14)	0.38	0.10	0.84
*Age-Accel*							
Grim-mAA	2.90E−11 (2.64)	− 1.35 (2.08)	1.15 (1.98)	2.29 (2.35)	**0.001**	** < 0.0001**	0.31
TL-mAA	− 1.61E−18 (0.14)	0.05 (0.13)	− 0.05 (0.13)	− 0.07 (0.11)	0.05	**0.008**	0.88
Horvath-mAA	1.45E−11 (3.72)	− 0.68 (3.21)	0.22 (4.52)	1.48 (3.89)	0.71	0.12	0.62

*NS* never-smokers, *EC* e-cig users, *SM* smokers

Tukey HSD adjusted *P* < 0.05 in bold

**Table 2:** Top ten canonical pathways for Grim-mAA-associated transcript genes

Ingenuity canonical pathways	*P*	Molecules
Neuroinflammation signaling pathway	1.59E−07	AKT3, CD40, CREB3L4, CX3CL1, CX3CR1, CXCL10, FOS, GABRB2, GAD1, HLA-DPB1, HLA-DQA1, HLA-DQA2, HLA-DQB1, HLA-DQB2, IL1B, MR1, MYD88, NFKB1, NOX1, PIK3CG, SLC1A2, SOD2, TGFB1, TLR4, TLR5, TLR7, TYROBP
Estrogen biosynthesis	1.68E−07	AKR1B15, AKR1C1/AKR1C2, AKR1C3, AKR1C4, CYP1B1, CYP2A6 (includes others), CYP4F8, CYP4X1, HSD17B11, HSD17B13
B cell development	2.48E−07	CD40, HLA-DPB1, HLA-DQA1, HLA-DQA2, HLA-DQB1, HLA-DQB2, IL7R, PTPRC, RAG2
ICOS-ICOSL signaling in T helper cells	1.02E−06	AKT3, CD4, CD40, HLA-DPB1, HLA-DQA1, HLA-DQA2, HLA-DQB1, HLA-DQB2, IL2RG, ITPR1, LCP2, NFKB1, NFKBIA, PIK3CG, PLEKHA2, PTEN, PTPRC, TRGV8
IL-17A signaling in airway cells	2.82E−06	AKT3, CCL20, CXCL1, CXCL3, MAP2K1, MUC5AC, NFKB1, NFKBIA, PIK3CG, PTEN, TRAF3IP2
PD-1, PD-L1 cancer immunotherapy pathway	4.27E−06	AKT3, CDK2, HLA-DPB1, HLA-DQA1, HLA-DQA2, HLA-DQB1, HLA-DQB2, IL2RG, LCP2, MR1, PIK3CG, PTEN, TGFB1
Granulocyte adhesion and diapedesis	9.19E−06	CCL20, CCR2, CLDN1, CLDN10, CX3CL1, CXCL1, CXCL10, CXCL16, CXCL2, CXCL3, CXCL9, IL1B, ITGA4, MSN, SDC4, SELP, SELPLG
NRF2-mediated oxidative stress response	9.40E−06	ABCC1, CBR1, CYP2A6 (includes others), DNAJC14, FOS, GCLC, GCLM, GPX2, GSR, GSTA4, JUNB, MAP2K1, MAP2K6, NQO1, PIK3CG, PRDX1, SOD1, SOD2, TXNRD1
PKCθ signaling in T lymphocytes	1.32E−05	CACNA1C, CACNA1D, CACNA2D3, CACNG4, CACNG6, CD4, FOS, HLA-DPB1, HLA-DQA1, HLA-DQA2, HLA-DQB1, HLA-DQB2, ITPR1, LCP2, MAP3K9, NFKB1, NFKBIA, PIK3CG, TRGV8
IL-17 signaling	1.32E−05	AKT3, CCL20, CXCL1, CXCL3, DEFB1, DEFB105A/DEFB105B, FOS, IL1B, MAP2K6, MUC5AC, NFKB1, PIK3CG, TGFB1, TNFSF13, TRAF3IP2, TRAF5, VEGFD

When it comes to mAA, SM and EC had a significant acceleration of Grim-mAA compared to NS (Tukey’s HSD test P < 0.0001 for SM and Tukey’s HSD test P = 0.001 for EC, Table 1, Fig. 1H). SM had a significant deceleration of TL-mAA compared to NS (Tukey’s HSD test P = 0.008), and EC had a borderline significant decelaration of TL-mAA compared to NS (Tukey’s HSD test P = 0.05, Table 1, Fig. 1I). Horvath-mAA was not significantly different between any groups (Table 1, Fig. 1J).

Given that mAA is a more biologically meaningful predictor than mAge as it is associated with age-related diseases independent of chronological age [24, 27], we investigated mAge-Accel for further analyses.

### Associations of smoking and vaping intensities on lung DNA methylation age accelerations

**Fig. 2:**
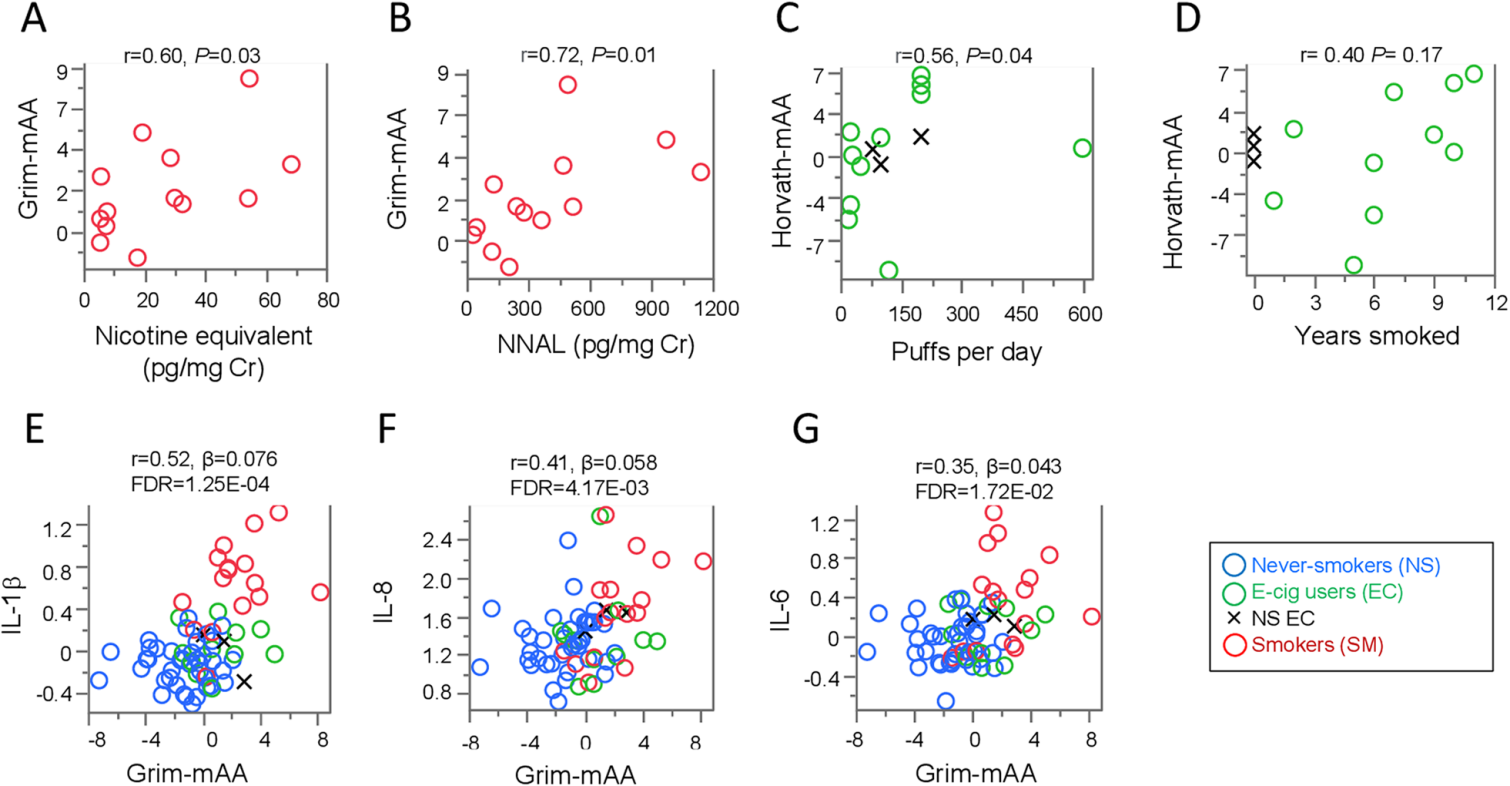
Relationships between Grim-mAA with urinary biomarkers, smoking indicators, and inflammatory biomarkers. Dot plots of correlations between **A** nicotine equivalent (Cotinine + 3-hydroxycotinine) and **B** NNAL (4-(methylnitrosamino)-1-(3-pyridyl)-1-butanol) with Grim-mAA (y-axis) among SM (red). Each open dot represents individual smokers. Dot plot of the correlation between **C** puffs per day (x-axis), **D** years smoked (x-axis), and Horvath-mAA (y-axis) among EC. Each open dot represents an individual electronic cigarette user. Black X’s represent never-smoking EC (NS EC). **E–G** Dot plots of associations between Grim-mAA (x-axis) and log_10_ transformed inflammatory cytokines (y-axis) displaying the partial correlation coefficient (r), effect size (β), and significant *P* value after adjusting for chronological age and gender including **E** IL-1β, **F** IL-8, and **G** IL-6. Each open dot represents an individual never-smoker (blue), electronic cigarette vaper (green) or cigarette smoker (red). Black X’s represent never-smoking EC

In SM, Grim-mAA was significantly positively correlated with nicotine equivalents (r = 0.60, P = 0.03, Fig. 2A) and 4-(methylnitrosamino)-1-(3-pyridyl)-1-butanol (NNAL, r = 0.72, P = 6.00E−03, Fig. 2B) (Additional file 3: Table 2).

For EC, puffs per day (Fig. 2C) were significantly positively correlated with Horvath-mAA (r = 0.56, P = 0.04), but no association was found with former smoking history (Fig. 2D) (Additional file 3: Table 2).

Propylene glycol (PG) levels were not significantly correlated with any mAA estimates. None of the aforementioned urinary biomarkers or smoking history indicators were significantly correlated with TL-mAA (Additional file 3: Table 2).

### Associations of lung DNA methylation acceleration with lung inflammatory cytokines and genome-wide gene expression

We further investigated overall lung inflammatory markers and genes to be associated with lung mAA estimates. Among ten cytokines measured, IL-1β (r = 0.52, β = 0.076, P = 1.30E−04), IL-8 (r = 0.41, β = 0.058, P = 4.20E−03), and IL-6 (r = 0.35, β = 0.043, P = 0.02) were each significantly positively associated with Grim-mAA independent of age and gender (Additional file 4: Table 3, Fig. 2E–G). There were no significant associations between any inflammatory cytokines and Horvath-mAA or TL-mAA.

**Fig. 3:**
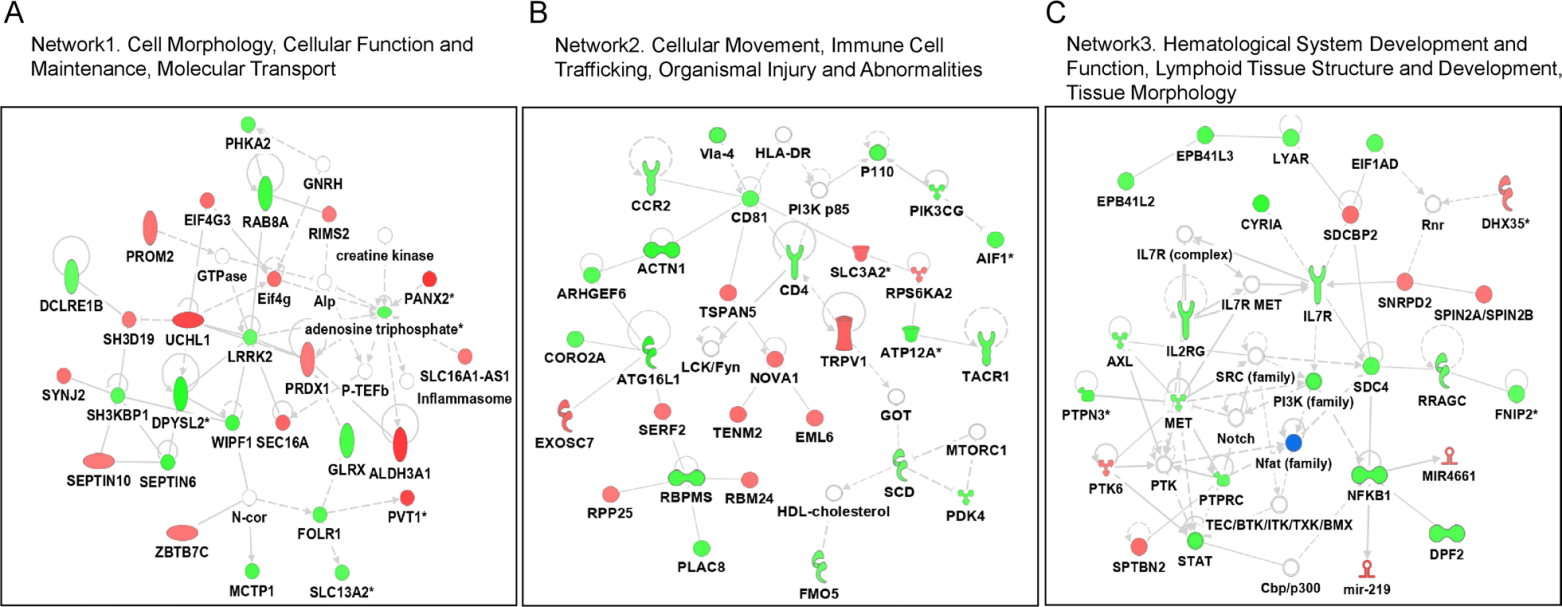
IPA top networks for Grim-mAA-associated genes. **A–C** The top three networks of genes significantly associated with Grim-mAA are displayed. Red molecules indicate genes that were positively correlated with Grim-mAA, and the green represents those that were negatively correlated. Nodal relationships are presented as solid lines, which represent direct interactions, and dashed lines, which indicate indirect interaction, as provided by ingenuity pathways analysis. The different functional classes of proteins are represented by varying shapes. A more in-depth description of the different shapes can be found at https://qiagen.secure.force.com/KnowledgeBase/articles/Basic_Technical_Q_A/Legend

For gene expression, we identified 759 transcripts (Additional file 5: Table 4) that were significantly associated with Grim-mAA, independent of gender and age (FDR < 0.1). None were associated with Horvath-mAA and TL-mAA. The majority of the transcripts (*n* = 626) were found to be significantly associated with smoking status (FDR < 0.1) (Additional file 6: Table 5). The top networks of the genes that were significantly correlate with Grim-mAA (Grim-mAA-associated genes) include cell morphology, cellular function and maintenance, molecular transport (network1), cellular movement, immune cell trafficking, organismal injury and abnormalities (network2), hematological system development and function, lymphoid tissue structure and development, and tissue morphology (network3) (Fig. 3). The top canonical pathways of these genes were highly enriched in immune-related pathways (Table 2).

### Potential involvement of genes that are associated with DNA methylation age acceleration in age-related pulmonary diseases

Given that Grim-mAA was significantly associated with inflammatory cytokines and expression of genes enriched in inflammation and the morphology and structures of cells/tissues, we explored the potential involvement of Grim-mAA-associated genes alterations in age-related lung diseases such as lung cancers and COPD.

**Fig. 4:**
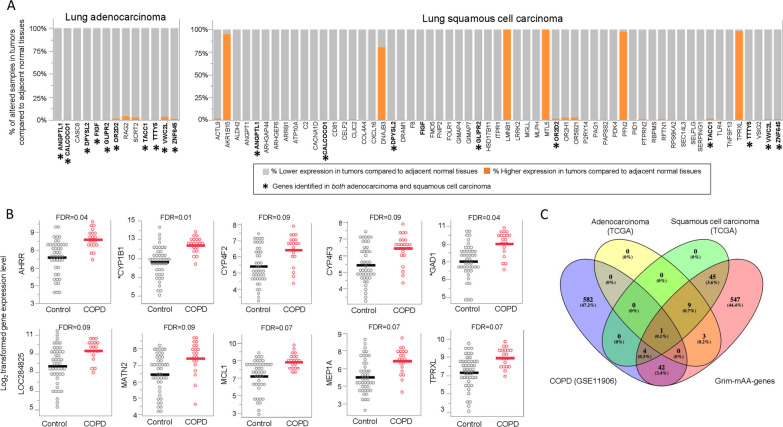
Differentially expressed Grim-mAA-associated genes in TCGA and COPD datasets.** A** Stacked bar charts display the percentage of altered samples with higher (orange) or lower (gray) expression in tumors compared to adjacent normal tissues (y-axis) for adenocarcinoma and squamous cell carcinoma by the gene (x-axis). Asterisks represent overlapping genes between adenocarcinoma and squamous cell carcinoma datasets. **B** Dot plots comparing the log_2_ expression (y-axis) between COPD cases (red) and controls (gray). Each open circle represents individual COPD cases and controls. Each line represents the mean log_2_ expression for each group. **C** Venn diagram of overlapping significant genes from TCGA, COPD data sets, and Grim-mAA-associated genes

For lung cancer in The Cancer Genome Atlas (TCGA) datasets, there were 618 genes of 651 unique Grim-mAA-associated genes available, 13 and 59 genes had significantly altered expression in ≥ 90% of lung adenocarcinoma samples and lung squamous cell carcinoma samples compared to their adjacent normal tissue, respectively (Fig. 4A, Additional file 7: Table 6). Ten genes were altered in both subtypes, including *ANGPTL1*, *CALCOCO1*, *DPYSL2*, *FIGF*, *GLIPR2*, *OR2D2*, *TACC1*, *TTTY5*, *VWC2L*, and *ZNF645*. Compared to adjacent normal tissue, these ten genes had lower expression in both subtypes (Fig. 4A). Some genes altered in either subtype are known to be involved in the cellular movement (*ALDH2*, *ANGPT1*, *RAG2*, and *TLR4*), cell morphology (*ALDH2*, *ARRB1*, *LRRK2*), and cell-to-cell signaling/interaction (*ANGPT1*, *CD81*, *TLR4*).

Of the 651 unique Grim-mAA-associated genes in the lungs of healthy individuals, 47 genes were significantly differentially expressed between COPD cases and healthy controls at FDR < 0.1 (Additional file 8: Table 7). Several genes had multiple transcripts for a gene (*n* = 3 for *CYP1B1*, *n* = 2 for *GAD1*, *n* = 2 for *TRIM7*, *n* = 2 for *VSIG10*), and all had higher expression in COPD compared to controls. Of the 47 genes altered in COPD cases, the top ten with the greatest fold changes were *AHRR*, *CYP1B1*, *CYP4F2*, *CYP4F3*, *GAD1*, *LOC284825*, *MATN2*, *MCL1*, *MEP1A,* and *TPRXL* (Fig. 4B). Figure 4C shows similarities and differences of genes between Grim-mAA-associated genes, TCGA, and COPD datasets. *TACC1* was identified to be different in lung adenocarcinoma, lung squamous cell carcinoma, and COPD patients.

## Discussion

This cross-sectional study of epigenetic aging in the lung revealed significant differences in comparisons of SM and EC to NS: more and faster aging, particularly Grim-mAge, and shorter and decelerated TL-mAge. It is generally considered that EC use is less toxic than smoking, including in the lung [12, 22, 37, 38]. However, this study indicates there may be some effect of EC on age-related pulmonary diseases.

Lung aging is an important risk factor for lung diseases, resulting in structural and physiological changes, including early carcinogenesis [39, 40]. In contrast to normal lung aging, we found that exposure to smoking or vaping was associated with accelerated lung aging. That aging may increase the rate of senescent cell accumulation and lung disease progression [3–5, 41]. mAge estimates have been predominantly examined in surrogate tissues, such as blood. Two recent studies focused on lung as the target organ, examining Grim-mAge and TL-mAge in lung epithelial tissue from those with HIV-associated COPD [42, 43]. However, there are, to our knowledge, no studies of mAge measures in the lungs of non-diseased individuals. Given our finding of significantly faster Grim-mAA and slower TL-mAA in SM and EC in a young adult cohort, these patterns could potentially be more accelerated in older populations. Also, because the duration of exposure to electronic cigarettes is necessarily short in young adults, we do not know the impact of more prolonged exposure on mAA.

While we observed significantly older Grim-mAge and shorter TL-mAge in healthy EC compared to NS, a conflicting finding was reported in saliva conducted in another study [22]. Differing duration of exposure to electronic cigarette between the studies (weekly vapers (ref) vs mostly daily vapers in our study) may explain this difference. Another possible explanation may be tissue type-specific DNA methylation differences (saliva vs lung) in epigenetic aging [20–22, 44]. Further, there may be some residual effects of former smoking among some of the EC. However, due to the limited sample size, we were not able to determine if there are unique EC effects. Determining such effects would require larger studies of EC users that include both those with and without former smoking.

Regarding smoking and epigenetic aging, consistent with previous blood studies [3, 24, 29–33], we observed significantly faster lung Grim-mAA in SM compared to NS, indicating that blood Grim-mAA may be a useful surrogate for aging in the lung. In a recent study of patients with COPD, blood Grim-mAA reflecting airway epigenetic age was suggested as a robust surrogate for airway epithelia aging [45]. Notably, we reported a possible effect of a tobacco-specific carcinogen (NNAL) on the rate of lung aging; to our knowledge, this report is the first of this association.

Altered inflammation is one of the major hallmarks of cancer [46], cell senescence-associated phenotype [47], and is associated with smoking and vaping [48]. While CpGs underlying Grim-mAge are known to be involved in cytokine-mediated signaling pathways [36], what cytokines are associated with altered Grim-mAA is unclear. We observed overall significant positive associations of Grim-mAA with the pro-inflammatory cytokines IL-1β, IL-8, and IL-6. These cytokines are known to be induced by increased reactive oxygen species (ROS) [49], are affected by smoking as reported by our group [12], and are associated with risk of lung cancer and lung diseases [48].

Separately, we identified several Grim-mAA-associated genes involved in immune-related gene pathways and in the morphology and structures of cells/tissues. Given that smoking and vaping generate ROS [50], it is plausible that faster lung epigenetic aging in SM and EC compared to NS affects the immune response and immune-related gene expression that may promote inflammation and related disease. However, further studies are needed to investigate the biology of this potential relationship and to understand the clinical relevance of our findings.

Of Grim-mAA-associated genes to be differentially expressed in lung adenocarcinoma and squamous cell carcinoma compared to their adjacent normal tissues, *ANGPTL1*, angiopoietin‑like protein 1, is a putative tumor suppressor in the lungs by repressing lung cancer cell motility [51]. *CALCOCO1* is an autophagy-associated protein and a transcriptional coactivator with *TCF/LET* and beta-catenin [52], but its role in lung cancer is unclear. *DPYSL2* is involved in tumor metastasis and has been elevated in smokers with COPD compared to never-smokers [53]. *TACC1* is involved in the process of transcription and translation [54], and found to be downregulated in LUAD compared to matched normal tissue [55]. Moreover, we found Grim-mAA-associated genes to be altered in COPD compared to controls. These identified genes with the greatest differences in expression are known to be associated with smoking, including gene families mediating the metabolism of xenobiotic substances [56], such as *AHRR*, *CYP1B1*, and *CYP4F3*. These genes are involved in oxidative stress upregulated by smoking exposure [57] and are associated with lung diseases such as COPD [58]. Additionally, *GAD1* in airway cells was found in COPD patients and is associated with increased epithelial *MUC5AC* [59]. Interestingly, we found several mucin families, including *MUC2*, *MUC5AC*, *MUC12*, and *MUCL1*, to be positively associated with Grim-mAA (Additional file 5: Table 4). Mucins are complex glycoproteins that are essential for protecting airways [60] and are typically induced by inhaled environmental insults such as smoking exposure and are associated with the initiation, promotion, and progression of COPD [61]. Mucins are overexpressed in *NSCLC* [62] and related to lung cancer prognosis [62, 63]. Although our findings do not provide a causal relationship between Grim-mAA and lung diseases, it may be plausible that smoking and vaping alter Grim-mAA-associated genes and increase susceptibility to respiratory diseases.

There are several strengths of our study. We examined lung tissue, providing evidence regarding direct target organ effects of smoking and vaping. The study subjects were young and would have less lung-related damage than older users. We investigated potential associations of mAA with exposure biomarkers for tobacco use and other lung biomarkers, allowing for a broad view of the potential effects of epigenetic aging on disease susceptibility. Further, we utilized publicly available datasets to understand the potential contribution of the observed mAA in age-related lung diseases.

However, it is also important to note the limitations of this study. As no lung tissue-specific mAge is available, estimated mAge may not reflect accurate biological aging. Thus, we focused on a relative comparison of mAge across the groups. Due to the cross-sectional nature of our study design, we cannot derive causal inferences. Consequently, our results require replication in prospective studies. Although we adjusted for potential confounding by chronological age and gender, other factors (e.g., occupation, social determinants of health-related variables, etc.) might need consideration. Additionally, EC reported a wide variety of devices, flavors, and nicotine concentrations, which each could have different effects which would influence our results. Due to the small sample size, we could not determine the impact of smoking or unique effect of EC on biological aging.

In summary, faster lung mAge for SM is consistent with prior studies in blood. Our finding of faster lung mAge for EC compared to NS indicates possible adverse pulmonary effects of EC on biological aging. Our findings support further research on the role of smoking and vaping in health and pulmonary diseases. Given that most EC are former smokers, further study is needed to uncover the unique effects of electronic cigarettes on biological aging.

## Methods

### Study participants

Participants, chronologically aged 21–30 years, were recruited (2015–2017) through the Ohio State University (OSU) Study Search and Research Match websites, local print, television media, and Craig’s List [12, 23, 37, 64]. Participants included EC vapers (EC; *n* = 14, including 3 never-smoking EC), smokers (SM; *n* = 16), and non-EC/non-SM (NS; *n* = 39) (Table 3**)**. The study protocol was approved by the OSU’s Institutional Review Board. Participants were classified as EC, SM, or NS as follows: EC were those who had been using nicotine-containing EC daily for ≥ 1 year and had not smoked a cigarette for > 6 months; SM were those who had used > 10 cigarettes per day for > 6 months and had not used an EC for ≥ 1 year; and NS were those who had smoked < 100 cigarettes in their lifetime (CDC Guideline) and had not used a cigarette or EC for ≥ 1 year before enrollment. Participants were excluded from the study if they had an immune system disorder requiring medication, pulmonary diseases, kidney or liver disease, or additional health conditions that would increase their risk from bronchoscopy or potentially influence biomarker outcomes. Participants were also excluded if they had: undergone general anesthesia, bronchoscopy or other lung procedure ≤ 12 months before the study; used inhalant medications; allergies to study medications; reported past usage of marijuana or combustible tobacco more than 10 times; used marijuana or other combustibles ≤ 3 months before the study; or, reported pregnancy.

**Table 3:** Characteristics of study participants

Cross sectional study (*n* = 69)	All subjects (*n* = 69)	Never-smokers (*n* = 39)	Electronic cigarette users (*n* = 14)	Smokers (*n* = 16)
Age, years, median (IQR)	26.5 (23.6–28.0)	25.7 (23.4–27.7)	27.2 (25.3–28.9)	26.3 (23.7–28.2)
*Gender*				
Females, N (%)	32 (46%)	24 (62%)	4 (29%)	4 (25%)
*Race*				
White, N (%)	55 (80%)	30 (77%)	11 (79%)	14 (88%)
Non-White, N (%)	14 (20%)	9 (23%)	3 (21%)	2 (13%)
*Smoking*				
Former, N (%)	–	–	11 (79%)	–
Current, N (%)	–	–	–	26 (100%)
Never, N (%)	–	43 (100%)	3 (21%)	–
Years of smoking, median (IQR)	–	–	6.5 (4.3–10.0)^a^	9.5 (4.0–10.0)
Pack Years, median (IQR)	–	–	3.7 (0.8–7.7)^a^	6.8 (2.9–10.0)
Cigarettes per day, median (IQR)	–	–	15 (2.8–20.0)^a^	20.0 (10.0–20.0)
Days since last cigarettes, median (IQR)	–	–	737.0 (316.5–1125.0)^a^	–
*Electronic cigarette (EC) use*				
Years of EC use, median (IQR)	–	–	3.0 (2.0–3.3)	–
Puffs per day, median (IQR)	–	–	100.0 (28.8–200.0)	–
EC–liquid (ml) per day, median (IQR)	–	–	9.0 (5.0–10.0)	–
Nicotine (mg/ml), median (IQR)	–	–	6.0 (3.0–13.5)	–
Urinary biomarkers*	–			
Nicotine equivalent (nmol/mg Cr), median (IQR)	–	0.003 (0.001–0.006)	12.1 (4.3–35.6)	19.5 (6.6–43.5)
Nicotelline (ng/mL), median (IQR)	–	–	2.0 (2.0–23.0)	1011.2 (124.2–1274.8)
Anatabine (ng/mL), median (IQR)	–	0.1 (0.1–0.1)	0.1 (0.1–2.0)	8.5 (2.5–19.8)
NNAL (pg/mg Cr), median (IQR)	–	0.5 (0.2–0.9)	12.4 (1.2–35.3)	278.1 (126.1–505.3)
Propylene glycol (mg/mL), median (IQR)	–	2.0 (0.9–5.0)	27.9 (5.5–54.2)	6.6 (2.7–20.8)

Never-smokers non-EC users/non-smokers. Nicotine equivalent [Cotinine + 3-hydroxycotinine]. NNAL [4-(methylnitrosamino)-1-(3-pyridyl)s-butanol]

*Cr* creatinine

*Below quantification limit was replaced by half of the limit of quantification

^a^Prior smoking EC users

### Bronchoscopy

Participants completed an orientation session and eligibility evaluation and provided informed consent before undergoing bronchoscopy. During a bronchoscopy, a bronchoalveolar lavage (BAL) and bronchial epithelial brushing of grossly normal airway epithelium from the main bronchus were conducted followed by OSU standards of care.

### Urinary biomarkers of exposure

Liquid chromatography–tandem mass spectrometry (LC–MS/MS) was performed as previously described [64, 65] for NNAL, nicotine equivalent, and PG.

### Inflammatory cytokines

Cell-free BAL fluid was assayed using the Meso Scale Discovery Sector Imager 2400A (Meso Scale Discovery, Rockville, MD) with a V-PLEX Plus Proinflam Combo 10 panel.

### Genome-wide DNA methylation and gene expression in lung tissues

Genome-wide DNA methylation and gene expression analyses in lung tissues include data from our previous study (32 out of 69)[12]. Samples for each assay were randomized by permuted block randomization. Batch effects were removed by ANOVA with the feature as the dependent variable and batch as the independent variable for both analyses. For genome-wide DNA methylation, bisulfite conversion of 500 ng of DNA was performed on each sample according to the manufacturer’s recommendations for the Infinium MethylationEPIC BeadChip (Illumina, San Diego, CA) on lung tissue samples collected via bronchoscopy. Raw data were transformed by subset-quantile within array normalization (SWAN) and logit-transformation of β-values to convert M-values for normality using Partek Genomics Suite™ 6.6 (St. Louis, MO). Probes with a detection P > 0.05 were excluded from the analysis.

GeneChip® Human Transcriptome Array 2.0 (Affymetrix Inc, Santa Clara, CA) was used for transcriptome analysis. CEL files were log_2_ transformed and underwent quantile normalization in Partek.

### Epigenetic age calculation

mAge estimates for DNA methylation-based chronological aging (Horvath-mAge) [35], lifespan and mortality risks (Grim-mAge) [36], and telomere length (TL-mAge) [31] were determined by using Horvath’s New Methylation Age Calculator (https://dnamage.genetics.ucla.edu/new) with Advanced Analysis [32]. SWAN normalized β-values were processed using the calculator’s internal normalization method. mAA estimates were obtained as the residuals calculated by a linear model of mAge on chronological age [35].

### Statistical analysis

All mAge and mAA estimates were normally distributed. mAge estimates were correlated with each other and with chronological age using Pearson’s correlation, and FDR < 0.05 for significance was considered. Between-group differences for mAge and mAA estimates were assessed using Tukey’s honestly significant difference (HSD), and P < 0.05 was reported as significant. Spearman correlations were used to correlate between smoking/EC variables and mAge estimates. Years smoked for never-smoking EC were considered 0. P < 0.05 was used as the cut-off for significance. To associate Grim-mAA with other biomarkers (cytokines and transcriptome), we used multiple regression adjusting for age and gender. Partial correlations and P-values were obtained from the models. Multiple testing adjustment was conducted within each analysis type (10 cytokines and 33,494 transcripts, separately), and FDR < 0.1 was considered significant.

### Ingenuity pathway analysis (IPA)

The canonical pathway analysis function included in IPA was used for mAA-associated genes to explore consistent empirical and biological relationships between genes. The significance of canonical pathways was determined by IPA’s default threshold [− log(*P* value) > 1.3], and we presented the top 10 most significant pathways at a significance level of < 10^–5^.

### Lung cancer and COPD public datasets

To investigate the potential association between mAA-associated genes and lung cancer, we utilized lung cancer datasets from TCGA. We used the PanCancer Atlas (*n* = 566 tumor and paired normal tissue for lung adenocarcinoma and *n* = 487 tumor and paired normal tissue for squamous cell carcinoma) using cBioPortal (https://www.cbioportal.org). mAA-associated genes from this study were investigated as z-scores calculated relative to matched adjacent normal tissue. A z-score of ≥ 2 or ≤ − 2 in any investigated genes was considered altered expression. We considered genes important if ≥ 90% of samples were altered. We also investigated expression data from GSE11906 and utilized the GEO2R web tool from GEO (Gene Expression Omnibus). We defined healthy smokers as “controls” and COPD samples as “cases” (20 cases and 44 controls) [66]. A FDR < 0.1 determined significantly different expressions between cases and controls.

### Supplementary Information

**Additional file 1**. **Supplementary Figure 1.** Histograms of biological aging estimates and smoothened density lines. Each solid line represents biological aging estimates (Grim-mAge, Horvath-mAge, and DNAmTL), for NS (blue), EC (green), or SM (red), while each dotted line represents chronological age for NS (blue), EC (green) or SM (red).

**Additional file 2**. **Supplementary Table 1.** Correlations between mAge and mAA measures.

**Additional file 3**. **Supplementary Table 2.** Spearman correlations of mAA with smoking history, electronic cigarette history and urinary biomakers.

**Additional file 4**. **Supplementary Table 3.** Relationships between Grim-mAA and inflammatory cytokines.

**Additional file 5**. **Supplementary Table 4.** List of transcripts significantly correlated with Grim-mAA at FDR < 0.1.

**Additional file 6**. **Supplementary Table 5.** Association of Grim-mAA associated transcripts with smoking status.

**Additional file 7**. **Supplementary Table 6.** Altered expression of Grim-mAA associated transcript genes in TCGA lung adenocarcinoma and squamous cell carcinoma samples compared to adjacent normal tissue.

**Additional file 8**. **Supplementary Table 7.** List of Grim-mAA associated genes identified in COPD data set (GSE11906).

## Data Availability

The data used to support this manuscript and all supplementary materials are reported in its entirety. Because of privacy and ethical issues, patient-level data cannot be reported. Readers may request to access this data for non-commercial use via email to the corresponding author, with an explanation of the detailed intended purposes for the data.
