# Supplementary material for: Accelerated epigenetic age, inflammation, and gene expression in lung: comparisons of smokers and vapers with non-smokers
Source: Clin Epigenetics. 2023 Oct 11;15:160. doi: 10.1186/s13148-023-01577-8 (PMC10568901; doi:10.1186/s13148-023-01577-8)
Supplement: Supplementary file 1 — Additional file 1. Supplementary Figure 1. Histograms of biological aging estimates and smoothened density lines. Each solid line represents biological aging estimates (Grim-mAge, Horvath-mAge, and DNAmTL), for NS (blue), EC (green), or SM (red), while each dotted line represents chronological age for NS (blue), EC (green) or SM (red). [file 13148_2023_1577_MOESM1_ESM.docx]

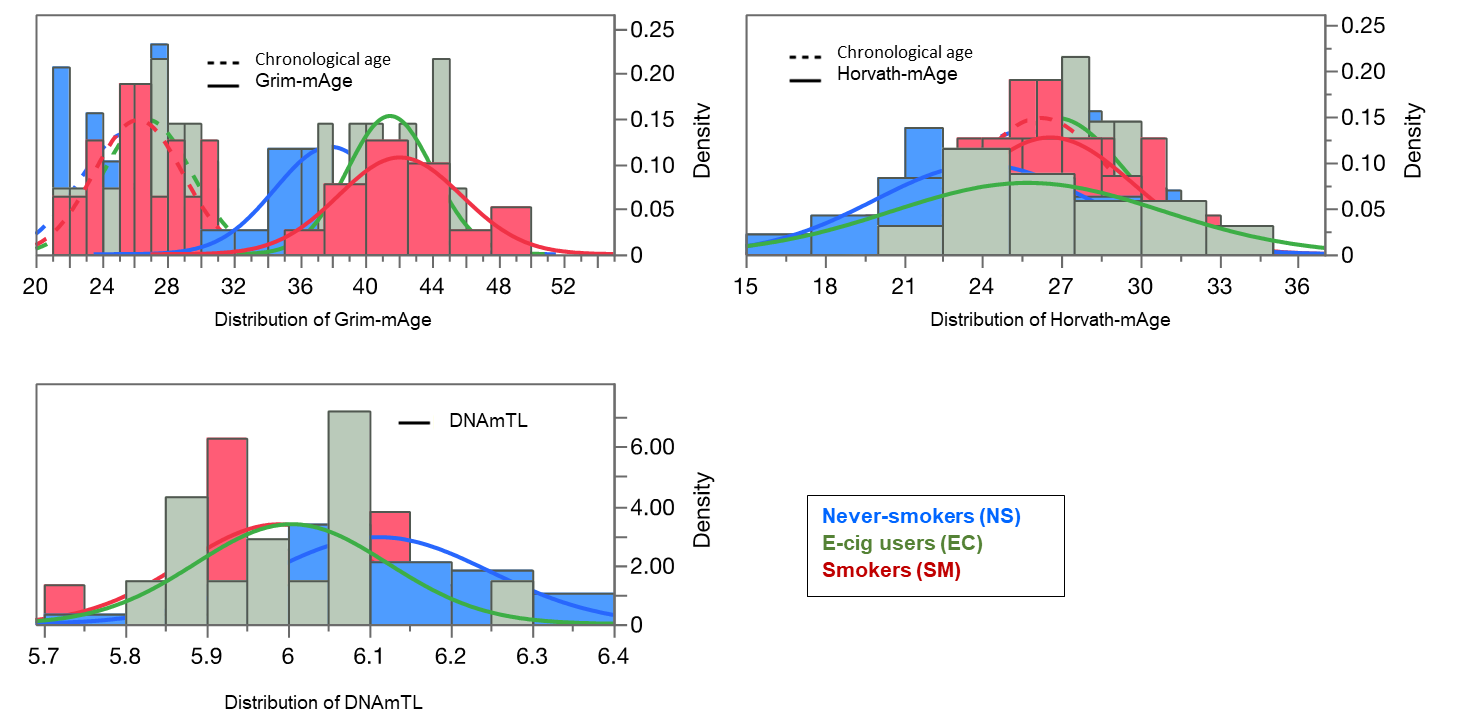


**Supplementary Figure 1.** Histograms of biological aging estimates and smoothened density lines. Each solid line represents biological aging estimates (Grim-mAge, Horvath-mAge, and DNAmTL), for NS (blue), EC (green), or SM (red), while each dotted line represents chronological age for NS (blue), EC (green) or SM (red).
